# A Phase 1 study of RO6870810, a novel bromodomain and extra-terminal protein inhibitor, in patients with NUT carcinoma, other solid tumours, or diffuse large B-cell lymphoma

**DOI:** 10.1038/s41416-020-01180-1

**Published:** 2020-12-14

**Authors:** Geoffrey I. Shapiro, Patricia LoRusso, Afshin Dowlati, Khanh T. Do, Caron A. Jacobson, Ulka Vaishampayan, Amy Weise, Paolo F. Caimi, Joseph Paul Eder, Christopher A. French, Emily Labriola-Tompkins, Frédéric Boisserie, William E. Pierceall, Jianguo Zhi, Sharon Passe, Mark DeMario, Martin Kornacker, Philippe Armand

**Affiliations:** 1 Department of Medical Oncology, Dana-Farber Cancer Institute, and Department of Medicine, Brigham and Women’s Hospital and Harvard Medical School, Boston, MA USA; 2 Early Phase Clinical Trials Program, Yale University Medical Center, New Haven, CT USA; 3 Department of Medicine-Hematology and Oncology, University Hospitals Seidman Cancer Center, Cleveland, OH USA; 4 Department of Oncology, Karmanos Cancer Institute, Detroit, MI USA; 5 Medical Oncology, Karmanos Cancer Institute, Detroit, MI USA; 6 Department of Pathology, Brigham and Women’s Hospital and Harvard Medical School, Boston, MA USA; 7 Roche Pharma Research and Early Development, Roche Innovation Center New York, New York, NY USA; 8 Roche Pharma Research and Early Development, Roche Innovation Center Basel, Basel, Switzerland

**Keywords:** Oncology, Targeted therapies

## Abstract

**Background:**

Bromodomain and extra-terminal (BET) proteins are epigenetic readers that can drive carcinogenesis and therapy resistance. RO6870810 is a novel, small-molecule BET inhibitor.

**Methods:**

We conducted a Phase 1 study of RO6870810 administered subcutaneously for 21 or 14 days of 28- or 21-day cycles, respectively, in patients with the nuclear protein of the testis carcinoma (NC), other solid tumours, or diffuse large B-cell lymphoma (DLBCL) with MYC deregulation.

**Results:**

Fatigue (42%), decreased appetite (35%) and injection-site erythema (35%) were the most common treatment-related adverse events. Pharmacokinetic parameters demonstrated linearity over the dose range tested and support once-daily dosing. Pharmacodynamic assessments demonstrated sustained decreases in CD11b levels in peripheral blood mononuclear cells. Objective response rates were 25% (2/8), 2% (1/47) and 11% (2/19) for patients with NC, other solid tumours and DLBCL, respectively. Responding tumours had evidence of deregulated MYC expression.

**Conclusions:**

This trial establishes the safety, favourable pharmacokinetics, evidence of target engagement and preliminary single-agent activity of RO6870810. Responses in patients with NC, other solid tumours and DLBCL provide proof-of-principle for BET inhibition in *MYC*-driven cancers. The results support further exploration of RO6870810 as monotherapy and in combinations.

**Clinical trials registration:**

NCT01987362.

## Background

Histone acetylation at *N*-terminal lysine residues is a common posttranslational modification that plays a major role in transcriptional regulation and contributes to other processes, such as DNA repair and replication.^1,2^ Protein interaction domains known as bromodomains (BRDs) recognise and bind to lysine acetylation motifs, thus allowing BRD-containing proteins to influence transcription.^3,4^ The BRD and extra-terminal (BET) family of proteins, which includes BRD2, BRD3, BRD4 and BRDT, have 2 *N*-terminal BRD modules in tandem (BD1 and BD2), an extra-terminal domain and several conserved motifs.^5,6^ BET proteins bind to acetylated chromatin via a hydrophobic pocket in the BRD modules and subsequently recruit and stabilise regulatory transcription effectors.^6,7^

BET proteins have been implicated in malignant transformation and in therapy resistance.^8^ Preclinical studies support the strategy of inhibiting the interaction between BET proteins and acetylated lysine to achieve anti-tumour effects. Direct-acting BET inhibitors utilise acetyl-lysine competitive binding to displace BET proteins from chromatin, resulting in preclinical activity in several solid tumour models, including those derived from colon, pancreatic and breast cancers.^4^ The selectivity of these compounds for transformed cells arises from the localisation of BET proteins to super-enhancers that regulate cell-specifying and oncogenic transcriptional programs, including those governed by the oncogene encoding the transcription factor c-MYC.^8^ In addition to MYC downregulation, BET inhibition has been shown to decrease oncogenic nuclear factor-κB activity, reduce ERK1/2 protein levels and decrease programmed death-ligand 1 and CXCL12 expression, thereby affecting immunity and cancer cell metastasis.^9–14^

Tumour types that may be particularly susceptible to BET inhibition include nuclear protein of the testis (NUT) carcinoma (NC) and diffuse large B-cell lymphoma (DLBCL).^5,15^ NC is a rare and aggressive type of poorly differentiated squamous cell carcinoma characterised by rearrangement in the *NUTM1* gene leading to the production of fusion oncoproteins, including BRD4-NUT, BRD3-NUT or NSD3-NUT.^16,17^ These fusion proteins have been shown to dysregulate *MYC*, which plays a key role in NC pathogenesis.^18^ In preclinical studies, BET inhibition displaced the BRD4-NUT oncoprotein from chromatin, resulting in anti-proliferative effects and squamous cell differentiation.^5^

In DLBCL, several key oncogenic pathways, including those driven by MYC- or E2F-dependent transcription and proliferation or Toll-like receptor signalling, are transcriptionally regulated by BET proteins.^15^ In preclinical models, BET inhibition impedes BRD binding to super-enhancer sites for MYC and other important transcription factors,^15,19,20^ thereby disrupting key lineage-specific transcriptional programs and resulting in anti-lymphoma activity. Because MYC deregulation in DLBCL is associated with aggressive clinical behaviour and poor outcomes, such as that seen in double-hit or double-expressor lymphomas,^21,22^ BET inhibition in these subtypes may be of particularly high therapeutic value, in addition to possible effects through disruption of other critical transcriptional pathways or modulation of microRNA expression.^23^

RO6870810 (RG6146; formerly TEN-010) is a novel small-molecule, noncovalent BET protein inhibitor. RO6870810 contains the same core thienodiazepine scaffold that interacts with BET family BRDs as one of the early and best-characterised BET inhibitors, JQ1,^5^ and was developed to improve on the low solubility, metabolic instability and interaction with serotonin receptors of JQ1 while maintaining biologic activity and efficacy. Alpha assay technology data suggest that RO6870810 has a high affinity for the acetyl-lysine recognition pocket of BET family bromodomains (BRD4, BRD3, BRD2 and BRDT) (data on file). Here we describe the findings from a Phase 1 study evaluating the safety, pharmacokinetics (PK), pharmacodynamics and clinical activity of RO6870810 in patients with solid tumours. In addition, independent cohorts of patients with NC and DLBCL harbouring *MYC* abnormalities were also evaluated based on their potential vulnerability to BET inhibition.

## Methods

### Study design and participants

This open-label, multicentre Phase 1 study (NP39141; NCT01987362) was conducted in two parts (Parts A and B). Patients in Part A received escalating doses of RO6870810 (0.03–0.65 mg/kg) in a standard 3 + 3 design to determine the maximum tolerated dose (MTD) and dose-limiting toxicities (DLTs) in patients with solid tumours. Part B (expansion cohort) was conducted to further characterise the safety and biologic effect of RO6870810 in a subset of patients with solid tumours. Two sub-studies in patients with NC and DLBCL were also conducted, with RO6870810 dosed at levels up to the MTD. Patients eligible for study inclusion had advanced solid malignancy, NC or advanced aggressive DLBCL with abnormal MYC expression (including protein overexpression or gene rearrangement). Any detectable MYC expression by immunohistochemistry (IHC) or gene rearrangement by fluorescence in situ hybridisation (FISH), based on local testing, was considered acceptable, without pre-specified thresholds. All indications were required to be histologically confirmed, progressive/persistent in nature and requiring treatment. Patients with a solid malignancy had to be refractory to or intolerant of standard treatments; patients with NC could have newly diagnosed or relapsed/refractory disease. Patients with advanced DLBCL had to have relapsed or progressed after ≥2 lines of therapy and not be eligible for curative therapy. DLBCL subtype was determined by local testing using the Hans algorithm based on IHC. The diagnosis of NC was based on ectopic expression of NUT protein by IHC and/or detection of *NUTM1* gene translocation by FISH. All patients were 18 years of age or older, had an Eastern Cooperative Oncology Group performance status ≤1 and had acceptable organ function. Full eligibility requirements, including exclusion criteria, are provided in the Supplementary Methods. This study was approved by local institutional review boards and conducted in accordance with the protocol, Good Clinical Practice standards and the Declaration of Helsinki. All enrolled patients gave written informed consent.

### Study treatment

RO6870810 was administered once daily by subcutaneous (SC) injection on days 1 to 21 of a 28-day cycle or on days 1–14 of a 21-day cycle. Treatment with RO6870810 was administered by a trained health professional during scheduled clinic visits. Following confirmation and documentation of appropriate self or caregiver dosing technique, all other doses in each cycle were administered in the clinic or at home. No dose modifications of any RO6870810 dose level were permitted during cycle 1. Additional information regarding dose modifications in later cycles is provided in the Supplementary Methods.

### Safety assessments

The primary objective of this study was to characterise the safety and tolerability of RO6870810. Patients were considered evaluable for safety if they received ≥1 injection of the study drug. All adverse events (AEs) were graded per the National Cancer Institute’s Common Terminology Criteria for Adverse Events (CTCAE) version 4.03.

DLTs were defined as AEs during cycle 1 that were at least possibly related to the study drug and met one of the following CTCAE criteria: grade 4 neutropenia lasting ≥5 days or grade 3 or 4 neutropenia with fever and/or infection; grade 4 thrombocytopenia (or grade 3 with bleeding); grade 4 anaemia; grade 3 or 4 non-haematologic toxicity (excluding grade 3 vomiting and grade 3 diarrhoea, including clinical sequelae such as electrolyte abnormalities occurring with suboptimal prophylactic and curative treatment with either toxicity and excluding alopecia); grade 3 or 4 skin ulceration or other skin and SC tissue disorders related to the SC injection of RO6870810; or dosing delay >14 days due to treatment-emergent AEs or related severe laboratory abnormalities. Grade ≤3 drug-related fever or skin or SC tissue disorders localised to the site of injection—including grade ≤3 rash, pruritus, skin induration or pain—were not considered DLTs.

The MTD was defined as the highest tested dose below the dose at which a DLT was observed in ≥2 patients. Data for patients with NC and lymphoma were evaluated but not included in the MTD determination.

### Efficacy assessments

Evaluation of the anticancer activity of RO6870810 was a secondary objective, as measured by time to first and best response, overall response rate, progression-free survival (PFS), duration of response and overall survival.

For patients with solid tumours, efficacy was assessed by radiological assessment or physical tumour measurements per Response Evaluation Criteria in Solid Tumours version 1.1.^24^ Radiological assessments (computed tomography (CT), magnetic resonance imaging and/or positron emission tomography (PET)/CT) were performed during the first visit of the screening period at the end of cycle 2 and then every two cycles thereafter. Patients with a response had a follow-up radiological assessment 4 weeks later for confirmation.

Response assessment in patients with DLBCL followed the Lugano classification.^25^ Radiological assessment consisted of a diagnostic CT at baseline and then every two cycles (standard of care) and combined PET-CT at baseline and then every four cycles or to confirm complete response (CR). Scans could also be obtained after one cycle following a study amendment.

Patients who received ≥1 injection of the study drug were considered evaluable for efficacy. Responders were defined as patients who achieved a confirmed CR or partial response (PR). PFS was defined as the time from the date of first study drug administration to the first date of objectively determined progressive disease (PD) or death from any cause. PFS was censored at the date of the most recent objective progression-free observation for patients who were still alive at the time of analysis and without evidence of tumour progression. For patients who received subsequent anticancer therapy prior to objective disease progression or death, PFS was censored at the date of the last objective progression-free observation prior to the date of subsequent therapy.

### PK parameters

The determination of RO6870810 PK was another secondary objective. RO6870810 plasma levels were measured during treatment and during the follow-up period. Venous blood samples were collected on multiple days during cycle 1 and on day 1 of subsequent cycles. Complete PK assessment parameters are included in the Supplementary Methods. Patients with sufficient data to determine PK parameters were included in the PK analysis population.

### Pharmacodynamics

For pharmacodynamic assessments, venous blood samples were collected and CD11b expression in monocytes was assessed as a surrogate marker of target engagement using flow cytometry before and after the first administration of RO6870810. Samples were obtained on cycle 1, day 1 (pre-dose and 2, 4 and 8 h post dose) and on cycle 1, day 15 (pre-dose and 2- and 4-h post dose). A single sample was also taken on days 1 and 15 in subsequent cycles for patients enrolled to cohorts utilising 28-day treatment cycles.

### Statistical methods

Formal hypothesis testing was not performed for this Phase 1 study. The sample size was based on a standard 3 + 3 dose-escalation design and was considered sufficient to evaluate the safety and clinical activity of RO6870810. AEs were coded using the current Medical Dictionary for Regulatory Activities, and safety and clinical efficacy were summarised using descriptive statistics. The 95% CI for the objective response rate was calculated using Wilson’s score method with continuity correction, and PFS was analysed using the Kaplan–Meier method. For the PK analyses, key exposure parameters such as maximum plasma concentration (*C*_max_) and area under the curve were summarised with descriptive statistics, and dose-exposure relationships were plotted graphically. Percentage changes in CD11b from baseline by flow cytometry were summarised with descriptive statistics and plotted over time. The relationship between percentage change from baseline in CD11b (average of three measurements taken pre-dose, at 2 h and at 4 h) on day 15 and the RO6870810 dose was plotted. To investigate the relationship between individual percentage changes in CD11b from baseline and RO6870810 concentration on day 15, the nonparametric regression LOESS curve fitting (locally weighted scatterplot smoothing) method was applied.

## Results

### Patient demographics and disposition

Seventy-four patients were enrolled between October 22, 2013, and October 19, 2016. The clinical cut-off date for analysis was August 30, 2017, at which time all 74 patients were evaluable for safety and efficacy. Patient demographics and baseline characteristics are shown in Table 1. Forty-seven patients had solid tumours other than NC, 8 had NC and 19 had DLBCL. Patients with solid tumours and DLBCL had received a median of 5 (min–max, 0–13) and 3 (min–max, 2–6) prior therapies, respectively. The most common solid tumour types enrolled were colorectal (23%), prostate (15%) and breast (11%).

**Table 1:** Patient demographics and baseline characteristics.

Characteristic	Solid tumours (*n* = 47)	NC (*n* = 8)	DLBCL (*n* = 19)
Median age (min–max), years	62 (26–84)	48 (33–62)	67 (49–82)
Male, *n* (%)	19 (40.4)	6 (75.0)	14 (73.7)
*ECOG PS,* n *(%)*^a^			
0	12 (25.5)	2 (25.0)	4 (21.1)
1	35 (74.5)	5 (62.5)	15 (78.9)
2	0	1 (12.5)	0
*Cancer type,* n *(%)*			
Colorectal Prostate Breast Ovarian Salivary gland Metastatic prostate NSCLC Pancreatic Adenocarcinoma pancreas CUP Endometrial Fallopian tube Gastrointestinal stromal Hepatic Oesophageal Sarcoma Squamous cell carcinoma Uterine NC DLBCL	11 (23.4) 7 (14.9) 5 (10.6) 4 (8.5) 4 (8.5) 2 (4.3) 2 (4.3) 2 (4.3) 1 (2.1) 1 (2.1) 1 (2.1) 1 (2.1) 1 (2.1) 1 (2.1) 1 (2.1) 1 (2.1) 1 (2.1) 1 (2.1) 0 0	0 0 0 0 0 0 0 0 0 0 0 0 0 0 0 0 1 (12.5)^b^ 0 7 (87.5) 0	0 0 0 0 0 0 0 0 0 0 0 0 0 0 0 0 0 0 0 19 (100)
Median number prior systemic therapies (min–max), *n*	5.0 (0–13)	1.0 (0–1)	3.0 (2–6)

*CUP* poorly differentiated carcinoma of unknown origin, *ECOG PS* Eastern Cooperative Group performance status.

^a^Patients were required to have an ECOG PS of 0 or 1; however, one patient with NC with an ECOG PS of 2 was considered study eligible.

^b^NC is a rare and aggressive genetically defined subtype of squamous cell carcinoma characterised by chromosomal rearrangement of the *NUT* gene.

Patients in Part A (*n* = 30) were enrolled in one of eight cohorts to receive RO6870810 SC at dose levels ranging from 0.03 to 0.65 mg/kg (Supplementary Fig. S1). Patients in the expansion cohort (Part B; *n* = 17) received RO6870810 at 0.65 mg/kg SC. RO6870810 was administered at dose levels from 0.1 to 0.65 mg/kg SC in the NC (*n* = 8) sub-study and at 0.3 or 0.45 mg/kg SC in the DLBCL (*n* = 19) sub-study. At data cut-off, one patient with NC remained on treatment and eight patients (solid tumours, *n* = 5; DLBCL, *n* = 3) underwent long-term follow-up.

### Summary of dose escalation

Overall, RO6870810 demonstrated tolerability across indications. A single DLT of grade 3 cholestatic hepatitis was reported in one patient with prostate cancer treated at 0.45 mg/kg on the 28-day schedule. This Part A cohort was expanded without additional DLTs, permitting dose escalation to 0.65 mg/kg on the 28-day schedule. No cycle 1 events qualified as a DLT at the 0.65-mg/kg dose level. However, treatment was not well tolerated, with discontinuations early in cycle 2 due to fatigue, suggesting marginal feasibility of a 21- of every 28-day schedule. Enrolment subsequently commenced in a 0.65-mg/kg cohort in which the drug was administered for 14 of every 21 days (Cohort 8). This dose was ultimately identified as the recommended phase 2 dose for solid tumours.

### Safety

All patients experienced ≥1 AE, with grade ≥3 events reported in 47%, 88% and 74% of patients with solid tumours, NC and DLBCL, respectively (Supplementary Table S1). The most common (occurring in ≥10% of all patients) treatment-emergent AEs are described in Supplementary Table S2. As shown in Table 2, the most common treatment-related AEs of any grade were fatigue (42%), decreased appetite and injection-site erythema (35% each), injection-site pain (32%) and nausea (31%). Fatigue (10%), thrombocytopenia (10%), anaemia (8%) and hyperbilirubinemia (5%) were the most frequently reported grade 3/4 treatment-related AEs. No treatment-related grade 5 AEs occurred. Three patients with solid tumours (6%), 1 with NC (13%) and 0 with DLBCL had ≥1 dose reduction, which was due to AEs. Dose omissions were reported in 24 (51%), 2 (25%) and 6 (32%) patients, respectively, with omissions due to AEs most commonly caused by fatigue, thrombocytopenia and anaemia.

**Table 2:** Treatment-related AEs that occurred in ≥ 10% (all grades) or in ≥ 1% (grade 3/4) of patients (*N* = 74).

Treatment-related AE	Events, *n* (%)	
	All grades	Grade 3/4
Any related AE	70 (94.6)	22 (29.7)
Fatigue	31 (41.9)	7 (9.5)
Decreased appetite	26 (35.1)	0
*AEs related to drug administration*		
Injection-site erythema	26 (35.1)	0
Injection-site pain	24 (32.4)	0
Injection-site induration	21 (28.4)	0
Injection-site pruritus	15 (20.3)	0
Injection-site reaction	10 (13.5)	0
Injection-site swelling	10 (13.5)	0
Nausea	23 (31.1)	1 (1.4)
Diarrhoea	19 (25.7)	0
Dysgeusia	19 (25.7)	0
Vomiting	15 (20.3)	1 (1.4)
Anaemia	12 (16.2)	6 (8.1)
Malaise	11 (14.9)	0
Thrombocytopenia	9 (12.2)	7 (9.5)
*AEs related to liver enzymes and metabolism*		
Blood bilirubin increased	9 (12.2)	4 (5.4)
ALT increased	4 (5.4)	1 (1.4)
AST increased	4 (5.4)	1 (1.4)
Bilirubin conjugated increased	3 (4.1)	2 (2.7)
Hepatic enzyme increased	1 (1.4)	1 (1.4)
Hepatitis	1(1.4)	1 (1.4)
Abdominal pain	3 (4.1)	1 (1.4)
Hyperkalaemia	3 (4.1)	1 (1.4)
Platelet count decreased	3 (4.1)	1 (1.4)
Blood glucose increased	2 (2.7)	1 (1.4)
*AEs related to dyspnoea*		
Dyspnoea exertional	2 (2.7)	1 (1.4)
Dyspnoea	1 (1.4)	1 (1.4)
Hypertension	2 (2.7)	1 (1.4)
Hyperuricemia	2 (2.7)	1 (1.4)
Cardiomyopathy	1 (1.4)	1 (1.4)
Cholestasis	1 (1.4)	1 (1.4)
Neutropenia	1 (1.4)	1 (1.4)
Peritoneal haemorrhage	1 (1.4)	1 (1.4)

*ALT* alanine aminotransferase, *AST* aspartate aminotransferase.

### Pharmacokinetics

**Fig. 1:**
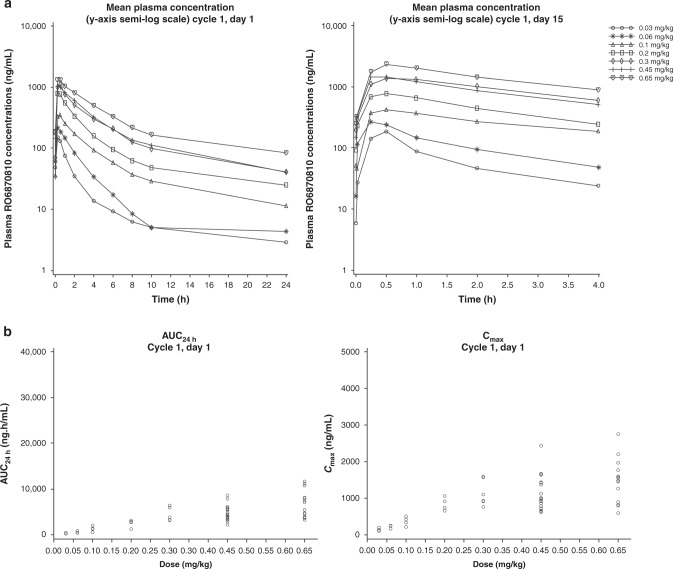
RO6870810 pharmacokinetic profile. **a** Plasma concentration-time profile on days 1 and 15 of cycle 1. **b** Dose-exposure relationship (cycle 1, day 1).

RO6870810 plasma concentration-time profiles on days 1 and 15 of cycle 1 for all patients are shown in Fig. 1a. RO6870810 demonstrated rapid absorption across dose levels. The *C*_max_ was generally achieved within 1 h (median, 0.5 h) of dosing on days 1 and 15 of cycle 1. The half-life was 10 h or longer. The dose-exposure relationship (*C*_max_ and area under the curve over 24 h) appeared dose-proportional across doses (Fig. 1b), and interpatient variability was moderate (38%) for both parameters. Clearance was found to be bodyweight dependent, and no metabolites were found in the leftover plasma samples screened.

### Pharmacodynamics

**Fig. 2:**
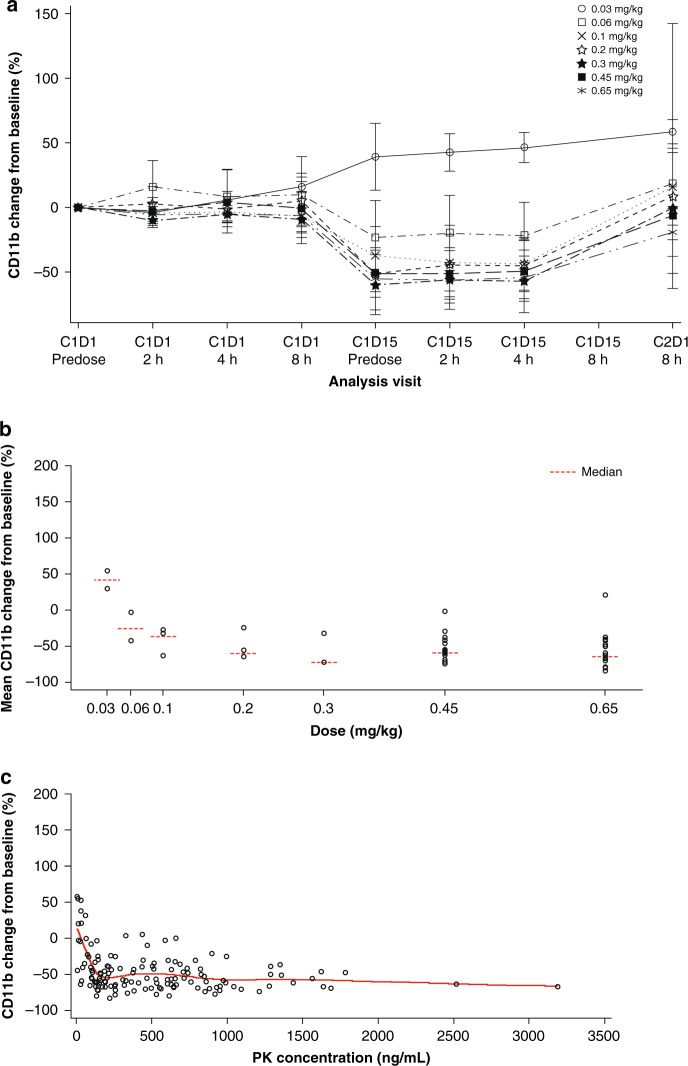
RO6870810 pharmacodynamic profiling as evidence of target engagement. **a** Mean (± SD) change in CD11b by RO6870810 dose on days 1 and 15 of cycle 1 and day 1 of cycle 2. **b** Median of mean change in CD11b by RO6870810 dose (cycle 1, day 15). Results are based on the average of three measurements per patient. **c** Change in CD11b by RO6870810 concentration and LOESS curve. Results are based on three observations per patient on cycle 1, day 15.

Chromatin immunoprecipitation sequencing previously identified a strong BRD4-driven super-enhancer near the *CD11b* promoter, and displacement of bound BRD4 from the super-enhancer element by a BET inhibitor results in diminished *CD11b* gene expression. In this study, CD11b expression on peripheral blood mononuclear cells was measured by flow cytometry pre-dose and at various time points between cycle 1 (day 1) and cycle 2 (day 1). Treatment with RO6870810 led to sustained decreases in CD11b during the dosing period across dose levels, with the exception of the 0.03-mg/kg dose (Fig. 2a). Mean decreases in CD11b occurred at doses >0.06 mg/kg, with the largest decreases (≥50%) observed at ≥0.2 mg/kg RO6870810 (Fig. 2b). These decreases were concentration-dependent, reaching a 50% reduction at concentrations >150 ng/mL (Fig. 2c).

### Clinical activity

**Fig. 3:**
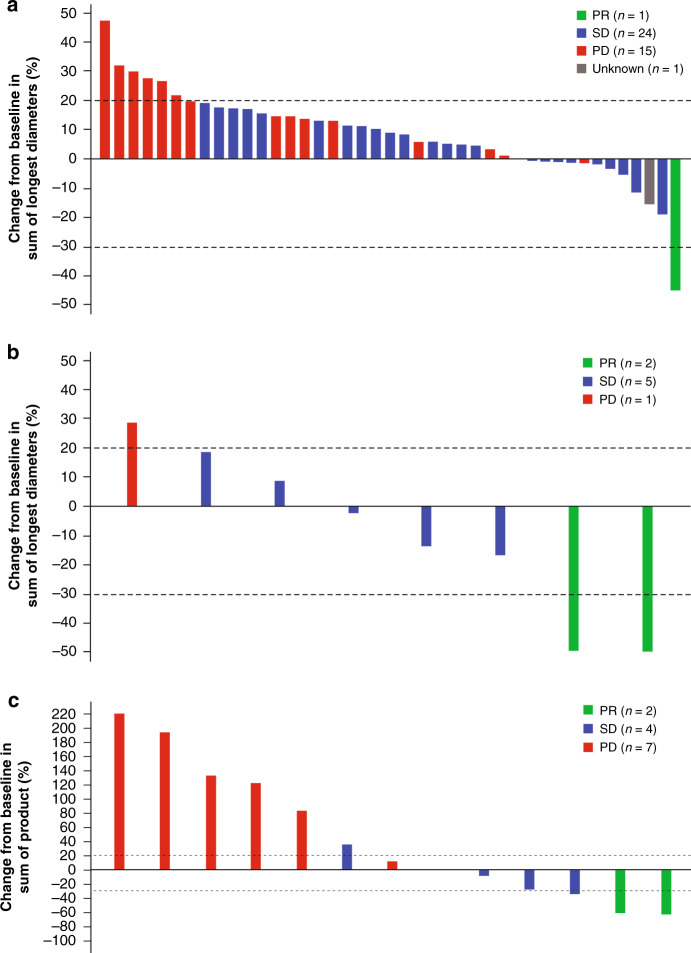
Changes from baseline to best response in measurements of target lesions. Percentage change from baseline to best response in target lesions is shown in patients with solid tumours (**a**), NC (**b**) and diffuse large B-cell lymphoma (DLBCL) (**c**). Only the largest decrease in the sum of the product or longest diameters for each eligible patient is shown in each figure. For patients with solid tumours, those with missing (*n* = 5) responses were excluded; one patient with prostate cancer who achieved SD as best response did not have a tumour diameter measurement and is not shown. Six patients with DLBCL without postbaseline tumour assessment were excluded. Among patients with solid tumours, a PR was reported in a patient with salivary gland cancer; SD was reported in patients with salivary gland cancer (*n* = 4), colorectal cancer (*n* = 5), oesophageal carcinoma (*n* = 1), non-small cell lung cancer (*n* = 1), prostate cancer (*n* = 7), breast cancer (*n* = 2), pancreatic cancer (*n* = 1), uterine cancer (*n* = 1), ovarian cancer (*n* = 2) and hepatic cancer (*n* = 1); PD was reported in patients with squamous cell carcinoma (*n* = 1), colorectal cancer (*n* = 4), non-small cell lung cancer (*n* = 1), poorly differentiated carcinoma of unknown origin (*n* = 1), pancreatic cancer (*n* = 1), breast cancer (*n* = 2), ovarian cancer (*n* = 1), gastrointestinal stromal tumour (*n* = 1), fallopian tube cancer (*n* = 1), endometrial cancer (*n* = 1) and adenocarcinoma pancreas (*n* = 1); and a patient with colorectal cancer had an unknown response.

Patients in the efficacy population are shown in Fig. 3 and Supplementary Table S3. Those with at least one postbaseline tumour assessment for whom response data were available are listed and include 8 of 8, 42 of 47 and 13 of 19 patients with NC, solid tumours and DLBCL, respectively.

#### NUT carcinoma

For the efficacy population, the objective response rate was 25% (95% CI, 4%–64%). A PR was achieved in 25% (*n* = 2) of patients, while 63% (*n* = 5) and 13% (*n* = 1) of patients had stable disease (SD) or PD as the best response, respectively. The median PFS among patients with NC was 94 days (range, 15–783 days). Clinical characteristics and outcomes for all 8 patients with NC are detailed in Supplementary Table S4.

Both patients who achieved PR had disease of thoracic origin (Supplementary Fig. S2). One of the responding patients with NC was a 54-year-old man who presented with a large right lower lobe mass extending to the hilum with extensive mediastinal adenopathy, liver metastases and extensive bone disease (Supplementary Table S4; patient 1). His tumour was positive for NUT expression by IHC and was found to harbour *BRD3-NUT* rearrangement. He had received no prior systemic therapy when he initiated treatment with RO6870810 at 0.45 mg/kg on 21 of every 28 days. His disease demonstrated regression after one cycle (‒26.4%) and PR after two cycles (‒49.3% at 52 days; Supplementary Fig. S2A). Dose delays and a reduction at cycle 3 to 0.3 mg/kg were required for AEs (thrombocytopenia and fatigue). After a subsequent modification to 0.45 mg/kg on the 14 of every 21-days schedule for cycle 4, PD was documented at day 115.

The second responding patient with NC was a 52-year-old man who also presented with an extensive thoracic primary tumour as well as rapidly progressive liver disease and bone metastases (Supplementary Table S4, patient 2). His tumour was found to be positive for NUT expression by IHC and to harbour *NSD3-NUT* fusion. He had previously received 6 cycles of etoposide and cisplatin but had residual disease. RO6870810 was initiated at 0.45 mg/kg for 21 of every 28 days. He transitioned to the schedule of 14 of every 21 days at cycle 2 and required dose delays in cycle 3 due to AEs, including fatigue and thrombocytopenia. He had a gradual reduction in measurable disease until a PR was documented during month 5 at day 160 (‒31.4%; Supplementary Fig. S2B). He did well until the 1-year mark (day 368) when CNS metastases developed. After whole-brain radiation, he resumed protocol treatment but continued to experience treatment delays, requiring a subsequent dose reduction to 0.3 mg/kg at cycle 17 (day 543). His nadir response was achieved during month 23 at day 711 (‒50%). He ultimately received 24 cycles of study treatment and discontinued therapy because of systemic PD during month 26 at day 797. Additional patients with NC also experienced a reduced tumour burden on RO6870810, although improvements were transient (Supplementary Fig. S3A, B).

#### Other solid tumours

Among patients in the solid tumour efficacy population, the objective response rate was 2% (*n* = 1 of 47; 95% CI, 0%–13%). Of the 42 patients with non-missing overall best response data, 2% achieved PR and 60% had SD as the best response; PD was reported in 36%. The median PFS for the 47 patients was 90 days (range, 9–315 days). The responding patient was a 73-year-old woman with metastatic acinic cell carcinoma of the parotid gland. She received primary surgery and chemotherapy treatment for metastasis, eventually experiencing disease progression. The primary and metastatic tumours demonstrated the somatic mutations *FBXW7 Q242H*, *FBXW7 S582L* as well as *KDM6A S763I* and *PIK3C2B Q877_W878K*, suggesting the possibility of bi-allelic *FBXW7* loss resulting in a high level of MYC protein expression, which was documented in the metastatic tumour (Supplementary Fig. S4A). She received RO6870810 at 0.45 mg/kg on 21 of every 28 days. Scans demonstrated regression after 1 cycle (‒25.3%) and a PR after two cycles (>40% reduction at 57 days and 46.8% reduction at nadir; Supplementary Fig. S4B). After treatment disruption due to AEs and a holiday, her disease progressed. She was permitted to resume treatment at 0.65 mg/kg on the schedule of 14 of every 21 days (cycle 4), and her disease responded once again. She experienced an elevation in troponin I, as well as a mild left ventricular ejection fraction reduction from a baseline of 65% to 45–50% with basolateral hypokinesis in the absence of angiographically significant coronary artery disease, possibly related to RO6870810. These events were considered to be cardiomyopathy by the investigator. The patient’s cardiac function partially recovered with medical intervention within 3–4 months off study treatment.

#### DLBCL

Among efficacy-evaluable patients with DLBCL, the objective response rate was 10.5% (*n* = 2 of 19; 95% CI, 2–35%). Of the 13 patients with non-missing overall best response data, 15% (*n* = 2) achieved PR, and 31% (*n* = 4) and 54% (*n* = 7) had SD or PD as best response, respectively. The median PFS for the 19 patients was 29 days (range, 18–708 days). Both responding patients were classified as having germinal centre B-cell (GCB) subtype DLBCL (Supplementary Fig. S5). The first was a 66-year-old man with stage IV disease with 20% to 30% MYC expression by IHC and extra copies of *MYC* without rearrangement as detected by FISH. He had received five prior lines of therapy: rituximab + cyclophosphamide + doxorubicin + vincristine + prednisone (R-CHOP); rituximab + ifosfamide + carboplatin + etoposide (R-ICE); dexamethasone + high-dose cytarabine + cisplatin (DHAP); rituximab + gemcitabine + oxaliplatin (R-GemOX); and rituximab + lenalidomide. RO6870810 was initiated at 0.45 mg/kg for 21 of every 28 days, and he achieved a PR (55% decrease in target tumour volume) after cycle 1 (Supplementary Fig. S5A). By the end of cycle 2, there was a 64% reduction from baseline in target tumour volume, although by objective criteria the patient had PD due to growth at other tumour sites. The patient proceeded to allogeneic stem cell transplant after four cycles of RO6870810 therapy.

The second patient was a 60-year-old man with hepatic involvement and 60% to 70% MYC expression and 50% to 70% BCL-2 expression by IHC, consistent with a double-expressing lymphoma. Prior to treatment with RO6870810, the patient had received R-CHOP, R-ICE and autologous stem cell transplant. He was treated with RO6870810 at 0.45 mg/kg for 14 of every 21 days and achieved a PR after cycle 1 (Supplementary Fig. S5B). At the end of cycle 2, PET scans showed increasing fluorodeoxyglucose (FDG) uptake of two hepatic lesions, consistent with PD.

## Discussion

Several BET inhibitors are currently under investigation for the treatment of solid tumours and haematologic malignancies.^4,8,26,27^ These agents have shown varying degrees of clinical activity and tolerability across indications.^28–34^ In this study, the BET inhibitor RO6870810 had an acceptable and manageable safety profile in patients with NC and other solid tumours and DLBCL, which was confirmed in an expansion cohort enrolled at the recommended phase 2 dose of 0.65 mg/kg administered for 14 of every 21 days. Toxicities encountered with RO6870810 demonstrated commonalities with those reported for orally administered birabresib (MK-8628; OTX015) and molibresib (GSK525762), such as fatigue, anaemia and thrombocytopenia, as well as gastrointestinal side effects, including diarrhoea, nausea, vomiting, reduced appetite, dysgeusia and increases in both indirect and direct bilirubin.^31,32^ The SC administration of RO6870810 did not prevent gastrointestinal toxicities in this study, suggesting that these AEs may represent on-target class effects.

While the reduced left ventricular ejection fraction that occurred in one patient temporally improved off treatment and was therefore possibly related to RO6870810, no other similar events occurred in the study population. Recent work has indicated that BET bromodomain inhibition abrogates adverse cardiac remodelling and fibrosis and prolongs survival in a model of genetic dilated cardiomyopathy by inhibiting the expression of pro-inflammatory gene networks in cardiac fibroblasts.^35^ These data support the assertion that the risk of RO6870810-induced cardiotoxicity is low. However, it should be noted that other preclinical studies have shown structural and functional alterations of cardiac mitochondria following BET inhibitor treatment.^36^

The incidence of treatment-related thrombocytopenia in our study (12%) was lower than that reported in studies of other BET inhibitors (22–96%).^28,31–34^ Though it is difficult to make direct comparisons due to differences in patient populations, there were no DLTs due to thrombocytopenia with RO6870810, which also differs from other agents in this class.^28,31–34^ While we cannot exclude insufficient exposure or target coverage as a potential explanation, RO6870810 did achieve pharmacokinetic exposures that were associated with pharmacodynamic effects. Interestingly, when RO6870810 monotherapy was administered in a Phase 1 study of patients with multiple myeloma (NCT03068351), using the same schedule at doses of 0.3, 0.45 and 0.6 mg/kg, grade 3–4 thrombocytopenia was observed in a substantial proportion of patients. However, in that study, it should be considered that bone marrow function in patients with multiple myeloma could be affected by the disease itself. Final results from the trial are currently being evaluated.

PK analyses revealed rapid absorption, linearity for exposure across the dose range tested, and bodyweight–dependent clearance, supporting a daily dosing strategy. The >10-h half-life of RO6870810 appears to be twice that of birabresib (4–5 h) and greater than that of molibresib (3–7 h).^31,32^

Decreases in CD11b in peripheral blood mononuclear cells, presumably the result of reduced *CD11b* gene expression, were observed with RO6870810 treatment, supporting the putative mechanism of action that RO6870810 prevents BET co-activator loading at super-enhancers. Reductions in CD11b correlated with plasma levels and were in general reversible after dosing ended during the treatment cycle. CD11b expression may serve as a pharmacodynamic marker that may help guide dosing and schedule optimisation in future studies.

RO6870810 showed clinical activity across indications, with two patients each in the NC and DLBCL sub-studies and one patient in the solid tumour cohort achieving PR. Interestingly, all of these patients likely had MYC-deregulated tumours, with high expression driven by *NUTM1* rearrangement, stabilised by *FBXW7* mutation or due to elevated *MYC* copy number or epigenetic mechanisms. These results validate preclinical predictions^9^ and suggest patient selection strategies that can be incorporated into future trials.

Both of the responding patients with NC had thoracic primary tumours, which are typically highly aggressive and rapidly fatal. Recently, a novel prognostic model of NC has confirmed the substantially shorter overall survival of thoracic compared with nonthoracic tumours, irrespective of the fusion partner expressed, whereas nonthoracic tumours may be subdivided, with longer survival documented among patients with tumours harbouring non-BRD4 fusions.^37^ Thus, in this study, clinical benefit was observed with BET inhibition among patients with NC carrying the worst prognosis.

Notably, the responding patients with NC had tumours harbouring BRD3-NUT and NSD3-NUT fusions, respectively. Interestingly, among patients with NC treated with molibresib, PRs (confirmed and unconfirmed) were mostly observed in tumours with BRD3-NUT fusions.^32^ Similarly, minor but sustained regression has also been reported in a patient with NC harbouring BRD3-NUT fusion treated with BMS-986158.^34^ Although all known NUT fusions depend on BRD3/BRD4 for their oncogenic function in NC, regardless of whether the NUT fusion partner is a BET protein, it is not known whether chromatin binding of BRD3-NUT and NSD3-NUT is more easily disrupted by competitive BET inhibition than that of BRD4-NUT. To this end, the patient with tumour harbouring NSD3-NUT fusion had a particularly striking course, remaining on study with a sustained PR for >2 years. Although all of the responding patients in this study received doses of 0.45 mg/kg, the response in this patient was sustained after dose reduction. Further preclinical biochemistry and clinical experience will be required to determine whether NCs harbouring such fusions are routinely amenable to BET inhibition. Although RO6870810 is not without biologic activity against BRD4-NUT fusions, where metabolic responses were observed, such responses did not translate to tumour regressions and were only transient.

The activity observed in patients with DLBCL is consistent with that of birabresib^28^ and CPI-0610^30^ in patients with relapsed or refractory disease. Among 22 patients with DLBCL treated with birabresib, 2 CRs and 1 PR have been reported—in both germinal and nongerminal centre B-cell–like subtypes. Of note, DLBCLs with c-MYC and BCL-2 or BCL-6 translocation, so-called double-hit lymphomas, as well as those with high surface expression of MYC and BCL-2 (double-expressor) are recognised to have poor prognosis,^22,38,39^ even if they do not necessarily display a coordinated set of other genetic disturbances.^40^ Both responding patients in this study had tumours with c-MYC expression, one of which was a double-expressor. Although the demonstration of activity in these patients is provocative, further assessment of patients in these DLBCL subsets will be required to determine whether c-MYC protein expression is the primary determinant of response to RO6870810. All patients in this study were required to have tumour harbouring some level of MYC expression or *MYC* translocation; however, despite this requirement, most patients did not demonstrate response. Similarly, only one of five patients with MYC-expressing DLBCL responded to treatment with birabresib.^28^ BET inhibition may modulate other pathways related to anti-apoptotic protein expression, nuclear factor-κB signalling, E2F-mediated gene expression or other super-enhancers of additional critical B-cell transcriptional factors that may also contribute to clinical activity.^15^

Across the study, many of the responses or incidences of disease regression or stabilisation tended to be brief. Preclinical models suggest multiple potential mechanisms of acquired resistance, including increased WNT or Hedgehog pathway signalling with β-catenin or GLI2-mediated MYC expression, respectively;^41–43^ hyperphosphorylation of BRD4 resulting in BRD-independent MED1 binding;^44^ kinome reprogramming;^45^ MAP kinase pathway activation;^46^ AMPK-ULK1–mediated autophagy;^47^ or upregulation of MCL1 or BCL-2.^48,49^ In future studies, post-progression biopsies will be valuable in defining potential mechanisms of resistance and in designing appropriate strategies to circumvent resistance. In this regard, RO6870810 is currently being evaluated in combination with venetoclax in patients with DLBCL (NCT03255096)—a strategy that may also be useful in double-hit or double-expressor lymphomas.

In conclusion, our findings demonstrate the safety and preliminary clinical activity of single-agent RO6870810 in solid tumours and DLBCL and provide a promising foundation for additional development, potentially in MYC-driven cancers. On the basis of preclinical studies, BET inhibition therapy may increase the efficacy and response durability associated with a variety of other anti-neoplastic agents providing for multiple combination strategies, including chemotherapy, histone deacetylase inhibitors and immunotherapeutics.^13,26,27^

## Supplementary information

Supplemental Data

## Data Availability

Qualified researchers may request access to individual patient level data through the clinical study data request platform (https://vivli.org/). Further details on Roche's criteria for eligible studies are available here (https://vivli.org/members/ourmembers/). For further details on Roche’s Global Policy on the Sharing of Clinical Information and how to request access to related clinical study documents, see here (https://www.roche.com/research_and_development/who_we_are_how_we_work/clinical_trials/our_commitment_to_data_sharing.htm).
