# Supplementary material for: A Phase 1 study of RO6870810, a novel bromodomain and extra-terminal protein inhibitor, in patients with NUT carcinoma, other solid tumours, or diffuse large B-cell lymphoma
Source: Br J Cancer. 2020 Dec 14;124(4):744–53. doi: 10.1038/s41416-020-01180-1 (PMC7884382; doi:10.1038/s41416-020-01180-1)
Supplement: Supplementary file 1 — Supplemental Data [file 41416_2020_1180_MOESM1_ESM.docx]

**Supplement to: Shapiro GI, LoRusso P, Dowlati A, Do KT, Jacobson CA, Vaishampayan U, et al. A Phase I Study of RO6870810, a Novel Bromodomain and Extra-Terminal Protein Inhibitor, in Patients with NUT Carcinoma, Other Solid Tumours, or Diffuse Large B-Cell Lymphoma**

**Supplem****ental Data**

*Supplementary methods*

*Additional inclusion criteria*

Patients were required to have:

- ≥1 metastatic tumour evaluable or measurable by radiographic imaging (patients with solid tumours)
- Life expectancy ≥3 months
- No active second/secondary or prior malignancies for ≥2 years, excluding currently treated basal cell or squamous cell carcinoma of the skin or carcinoma in situ of the cervix or breast
- Acceptable liver function, defined as bilirubin ≤1.5 times the upper limit of normal (ULN) and aspartate aminotransferase, alanine aminotransferase, and alkaline phosphatase ≤2.5 × ULN (if liver metastases were present, then ≤5 × ULN was allowed)
- Acceptable renal function, defined as calculated creatinine clearance ≥50 mL/min using the Cockcroft and Gault Method
- Acceptable haematologic status, which was defined as a granulocyte count ≥1500 cells/mm^3^, haemoglobin ≥9 g/dL (with or without transfusion), and platelet count
  ≥100,000 (platelets/mm^3^). For patients with diffuse large B-cell lymphoma (DLBCL) only: If the CTC grade 1 platelet count was ≥75,000 (platelets/mm^3^) and <100,000 (platelets/mm^3^), the patient could be enrolled with approval of the Medical Monitor
- Acceptable coagulation status, which was defined as prothrombin time/partial thromboplastin time ≤1.2 × ULN (unless receiving anticoagulation therapy) and international normalised ratio (INR) ≤1.6 (unless receiving anticoagulation therapy). For patients receiving anticoagulation therapy, eligibility was based on the INR. For patients receiving warfarin, acceptable coagulation status was defined as an INR ≤ 3.0 and no active bleeding
- Patients (women of childbearing potential and men) also agreed to use adequate contraception during the study and from 1 month prior to their first dose to 4 months after their last dose of study drug. If a woman or female partner of a man became pregnant or suspected pregnancy while participating in the study, he or she was required to inform the study physician immediately
- Patients also had to be reliable and willing to make themselves available for the duration of the study, to follow study procedures, and to provide written informed consent prior to any study-specific procedures

*Full list of exclusion criteria*

Patients meeting any of the following general criteria were ineligible for study entry:

- Haematologic malignancies
- New York Heart Association Class III or IV cardiac disease, myocardial infarction within the past 6 months, unstable arrhythmia
- Fridericia-corrected QT interval >470 msec (female) or >450 msec (male) or a history of congenital long QT syndrome. Any electrocardiogram abnormality, including pericarditis, that in the opinion of the Investigator would preclude safe participation in the study
- Active, uncontrolled infections requiring systemic therapy within 7 days of study entry
- Known, clinically important respiratory impairment (eg, diagnosis of obstructive lung disease, including patients with forced expiratory volume in the first 1 s of expiration [FEV_1_] <60% of the predicted value; diagnosis of restrictive lung disease, including patients with total lung capacity [TLC] <60% of predicted value or history of idiopathic pulmonary fibrosis). In patients with a pulmonary resection or lung tumour, an assessment of respiratory impairment using predicted values for FEV_1_ and TLC were not representative of normal function; in these specific circumstances, evaluation of respiratory impairment was determined by the Investigator and Medical Monitor
- Positive for human immunodeficiency virus, hepatitis B surface antigen, or hepatitis C antibodies
- History of major organ transplant
- History of an autologous or allogeneic bone marrow transplant. Patients with DLBCL may have had a previous autologous transplant but not within 90 days of study entry
- Symptomatic central nervous system malignancy or metastasis
- Serious nonmalignant disease that could compromise protocol objectives in the opinion of the investigator and/or the sponsor
- Pregnant or nursing
- Unwillingness or inability to comply with procedures required in this protocol
- Active substance abuse

Medication-related exclusion criteria:

- Prior treatment with small-molecule bromodomain and extra-terminal (BET) family inhibitor
- Significant allergy to a biological pharmaceutical therapy that poses an increased risk to the patient, in the opinion of the investigator
- Radiation for symptomatic lesions completed ≥14 days prior to study entry. Patients with treated central nervous system metastases were eligible provided their disease was asymptomatic and that they were not currently receiving corticosteroids and/or anticonvulsants
- Patients with advanced solid tumours:
  - Treatment with surgery or chemotherapy within 28 days prior to study entry (42 days for nitrosoureas or mitomycin C)
  - Currently receiving any other investigational agent or received an investigational agent within 30 days or 5 half-lives prior to study entry, whichever was longer
- Patients with nuclear protein of the testis (NUT) carcinoma (NMC):
  - Treatment with surgery, other investigational agent, or chemotherapy within 14 days prior to study entry

*Study participation and treatment*

Study participation included a 28-day screening period, a treatment period consisting of repeating 21- or 28-day treatment cycles, an end-of-treatment visit conducted 30 days posttreatment, and long-term follow-up. Patients were monitored per routine clinical practice during the long-term follow-up period until disease progression or death. Patients who discontinued study treatment without documented disease progression were evaluation for tumour response per Response Evaluation Criteria in Solid Tumours version 1.1 (RECIST v1.1) or Lugano classification until disease progression, death, or study closure—whichever occurred first.

Dose modifications were not permitted in cycle 1 of Part A or without prior permission from the sponsor in Part B. After cycle 1, dose modifications were permitted at the discretion of the Investigator or following consultation with the sponsor. Dose escalations were only allowed at the beginning of a treatment cycle, and patients in Part A who experienced a dose-limiting toxicity (DLT) during cycle 1 were withdrawn from the study. If a patient in Part A or B experienced a DLT-equivalent toxicity (cycles 2 or beyond), treatment was interrupted until the toxicity returned to grade ≤1 (nonhaematologic, except alopecia, fatigue, skin rash, nausea, vomiting, or diarrhoea that can be controlled with concomitant medications) or baseline (haematologic and nonhaematological if present at study entry); treatment could be resumed at the next lower dose level. After the first occurrence of a toxicity necessitating study treatment withholding, patients could restart RO6870810 at the cohort-identified dose after the toxicity resolved to grade ≤1 or baseline. No dose reductions of RO6870810 were permitted during cycle 1 of any dose level in Part A. Patients enrolled in cohort 1 of Part A could have a dose level reduction to 0.01 mg/kg after cycle 1.

*Pharmacokinetic assessments*

RO6870810 plasma concentrations were assessed in blood samples collected as follows:

Cycle 1:

- Cycle 1, day 1: Blood samples were collected before and immediately after injection and 0.25, 0.5, 1, 2, 4, 6, 8, and 10 hours after the injection was completed
- Cycle 1, day 2: Blood samples were collected 24 hours (pre-dose) and 28 hours (4 hours after receiving a second dose) after the injection on day 1 was completed
- Cycle 1, day 8: A blood sample was drawn at the visit as a random value
- Cycle 1, day 15: Blood samples were drawn before and immediately after injection and 0.25, 0.5, 1, 2, and 4 hours after the injection was completed
- Cycle 1, day 22: Blood samples were drawn at the visit as a random value (28-day treatment cycle only)

Cycle 2 and beyond:

- Cycle X, day 1: On day 1 of all cycles after cycle 1, blood samples were drawn as a random value during the study visit

End of treatment:

- A blood sample was drawn as a random value during the end-of-treatment visit on early termination

**Table S1. Safety summary**

| Event, n (%) | Solid tumours  (n = 47) | NC  (n = 8) | DLBCL  (n = 19) |
| --- | --- | --- | --- |
| Any AE^a^ | 47 (100.0) | 8 (100.0) | 19 (100.0) |
| Grade ≥3 AE | 22 (46.8) | 7 (87.5) | 14 (73.7) |
| Any treatment-related AE | 46 (97.9) | 6 (75.0) | 18 (94.7) |
| Grade ≥3 treatment- related AE | 11 (23.4) | 4 (50.0) | 7 (36.8) |
| SAEs | 13 (27.7) | 6 (75.0) | 10 (52.6) |
| Treatment-related SAEs | 5 (10.6) | 0 | 2 (10.5) |
| Treatment discontinuation  due to AE | 7 (14.9) | 0 | 1 (5.3) |
| Treatment discontinuation  due to treatment-related AE | 6 (12.8) | 0 | 1 (5.3) |
| Study discontinuation  due to AE | 4 (8.5) | 2 (25.0) | 3 (15.8) |
| DLT | 1 (2.1) | 0 | 0 |
| Death due to AE | 2 (4.3) | 3 (37.5) | 3 (15.8) |
| Death due to  treatment-related AE | 0 | 0 | 0 |

^a^ Grade 5 AEs were ventricular fibrillation (colorectal cancer, 0.1 mg/kg), respiratory failure (breast cancer, 0.65 mg/kg; NC [n = 2], 0.45 mg/kg), haemorrhagic pneumonia (DLBCL, 0.45 mg/kg), pneumonia and respiratory failure (DLBCL, 0.45 mg/kg), and encephalopathy (DLBCL, 0.45 mg/kg). None of these events were considered to be related to the study drug. Abbreviations: AE, adverse event; SAE, serious adverse event.

**Table S2. Treatment-emergent AEs occurring in ≥ 10% of all patients**

| AE, n (%) | 28-Day Cycles | | | | | | | 21-Day Cycles | | Overall  N = 74 |
| --- | --- | --- | --- | --- | --- | --- | --- | --- | --- | --- |
|  | **Cohort 1**  **0.03 mg/kg n = 3** | **Cohort 2**  **0.06 mg/kg n = 3** | **Cohort 3**  **0.1 mg/kg n = 4** | **Cohort 4**  **0.2 mg/kg n = 4** | **Cohort 5**  **0.3 mg/kg n = 6** | **Cohort 6**  **0.45 mg/kg**  **n = 15** | **Cohort 7**  **0.65 mg/kg n = 4** | **Cohort 7**  **0.45 mg/kg n = 14** | **Cohort 8**  **0.65 mg/kg n = 21** |  |
| Fatigue | 0 | 2 (66.7) | 3 (75.0) | 2 (50.0) | 2 (33.3) | 9 (60.0) | 3 (75.0) | 8 (57.1) | 14 (66.7) | 43 (58.1) |
| Decreased appetite | 0 | 0 | 4 (100) | 3 (75.0) | 6 (100) | 4 (26.7) | 2 (50.0) | 6 (42.9) | 13 (61.9) | 38 (51.4) |
| Nausea | 1 (33.3) | 0 | 2 (50.0) | 2 (50.0) | 3 (50.0) | 9 (60.0) | 1 (25.0) | 6 (42.9) | 8 (38.1) | 32 (43.2) |
| Vomiting | 0 | 0 | 4 (100) | 2 (50.0) | 2 (33.3) | 6 (40.0) | 1 (25.0) | 5 (35.7) | 8 (38.1) | 28 (37.8) |
| Diarrhea | 1 (33.3) | 0 | 3 (75.0) | 1 (25.0) | 2 (33.3) | 5 (33.3) | 2 (50.0) | 4 (28.6) | 8 (38.1) | 26 (35.1) |
| AEs related to drug administration | | | | | | | | | | |
| Injection  site erythema | 2 (66.7) | 1 (33.3) | 2 (50.0) | 3 (75.0) | 3 (50.0) | 7 (46.7) | 2 (50.0) | 2 (14.3) | 4 (19.0) | 26 (35.1) |
| Injection  site pain | 0 | 1 (33.3) | 1 (25.0) | 2 (50.0) | 2 (33.3) | 10 (66.7) | 3 (75.0) | 3 (21.4) | 3 (14.3) | 25 (33.8) |
| Injection  site induration | 1 (33.3) | 0 | 3 (75.0) | 2 (50.0) | 2 (33.3) | 7 (46.7) | 2 (50.0) | 1 (7.1) | 3 (14.3) | 21 (28.4) |
| Injection  site pruritus | 3 (100) | 1 (33.3) | 0 | 1 (25.0) | 2 (33.3) | 4 (26.7) | 1 (25.0) | 0 | 3 (14.3) | 15 (20.3) |
| Injection  site reaction | 0 | 0 | 0 | 0 | 0 | 1 (6.7) | 0 | 2 (14.3) | 7 (33.3) | 10 (13.5) |
| Injection  site swelling | 1 (33.3) | 0 | 1 (25.0) | 2 (50.0) | 2 (33.3) | 3 (20.0) | 0 | 1 (7.1) | 0 | 10 (13.5) |
| Anemia | 0 | 0 | 2 (50.0) | 0 | 2 (33.3) | 8 (53.3) | 1 (25.0) | 1 (7.1) | 6 (28.6) | 20 (27.0) |
| Dysgeusia | 0 | 0 | 0 | 1 (25.0) | 1 (16.7) | 4 (26.7) | 1 (25.0) | 3 (21.4) | 10 (47.6) | 20 (27.0) |
| Malaise | 1 (33.3) | 1 (33.3) | 1 (25.0) | 1 (25.0) | 1 (16.7) | 3 (20.0) | 0 | 4 (28.6) | 4 (19.0) | 16 (21.6) |
| Blood bilirubin increased | 0 | 0 | 1 (25.0) | 1 (25.0) | 1 (16.7) | 6 (40.0) | 1 (25.0) | 2 (14.3) | 2 (9.5) | 14 (18.9) |
| Constipation | 0 | 1 (33.3%) | 3 (75.0) | 1 (25.0) | 0 | 4 (26.7) | 1 (25.0) | 0 | 4 (19.0) | 14 (18.9) |
| Abdominal pain | 1 (33.3) | 1 (33.3) | 1 (25.0) | 1 (25.0) | 1 (16.7) | 4 (26.7) | 1 (25.0) | 1 (7.1) | 2 (9.5) | 13 (17.6) |
| Back pain | 0 | 0 | 2 (50.0) | 1 (25.0) | 0 | 4 (26.7) | 0 | 4 (28.6) | 2 (9.5) | 13 (17.6) |
| Dehydration | 0 | 0 | 2 (50.0) | 0 | 2 (33.3) | 3 (20.0) | 0 | 2 (14.3) | 4 (19.0) | 13 (17.6) |
| Dyspnea | 0 | 0 | 1 (25.0) | 2 (50.0) | 0 | 4 (26.7) | 1 (25.0) | 3 (21.4) | 1 (4.8) | 12 (16.2) |
| Headache | 0 | 0 | 1 (25.0) | 1 (25.0) | 3 (50.0) | 1 (6.7) | 0 | 3 (21.4) | 3 (14.3) | 12 (16.2) |
| Myalgia | 1 (33.3) | 0 | 1 (25.0) | 0 | 3 (50.0) | 3 (20.0) | 0 | 1 (7.1) | 3 (14.3) | 12 (16.2) |
| Dry mouth | 0 | 0 | 1 (25.0) | 1 (25.0) | 2 (33.3) | 3 (20.0) | 1 (25.0) | 1 (7.1) | 2 (9.5) | 11 (14.9) |
| Cough | 0 | 0 | 1 (25.0) | 0 | 0 | 3 (20.0) | 0 | 3 (21.4) | 3 (14.3) | 10 (13.5) |
| Dizziness | 0 | 0 | 2 (50.0) | 1 (25.0) | 1 (16.7) | 2 (13.3) | 0 | 2 (14.3) | 2 (9.5) | 10 (13.5) |
| Hypomagnesaemia | 1 (33.3) | 0 | 0 | 0 | 1 (16.7) | 2 (13.3) | 1 (25.0) | 0 | 5 (23.8) | 10 (13.5) |
| Thrombocytopenia | 0 | 0 | 0 | 0 | 1 (16.7) | 4 (26.7) | 0 | 2 (14.3) | 3 (14.3) | 10 (13.5) |
| Weight decreased | 1 (33.3) | 0 | 0 | 0 | 2 (33.3) | 2 (13.3) | 1 (25.0) | 1 (7.1) | 2 (9.5) | 9 (12.2) |

**Table S3. Summary of response rate (efficacy population)**

|  | Solid tumours  (n = 47) | NC  (n = 8) | DLBCL  (n = 19) |
| --- | --- | --- | --- |
| Objective response rate (95% CI), %^a,b^ | 2.1  (0%–0.13%) | 25.0  (0.04%–0.64%) | 10.5  (0.02%–0.35%) |
| Complete response, n (%) | 0 | 0 | 0 |
| Partial response, n (%) | 1 (2.4)^c^ | 2 (25.0) | 2 (15.4)^d^ |
| Stable disease, n (%) | 25 (59.5)^c^ | 5 (62.5) | 4 (30.8)^d^ |
| Progressive disease, n (%) | 15 (35.7)^c^ | 1 (12.5) | 7 (53.8)^d^ |
| Unknown, n (%)^e^ | 1 (2.4)^b^ | 0 | 0 |
| Missing, n (%)^f^ | 5 | 0 | 6 |
| Median time to first response  (min-max), days | 56.0 | 105.5 (52–159) | 45.0 (41–49) |

^a^ Objective response rate is defined as the number of responders divided by the number of patients qualified for tumor response analysis (i.e., those who received ≥1 injection). 95% CI is based on Wilson’s score method with continuity correction.

^b^ A responder is defined as any patient who exhibits a complete response or partial response. A patient with a missing or unknown response was assumed to be a non-responder.

^c^ For these percentage calculations, n = the number of patients with non-missing overall best response data, n = 42.

^d^ For these percentage calculations, n = the number of patients with non-missing overall best response data, n = 13.

^e^ Responses were considered unknown when all target lesions were not evaluated (1 patient, 0.1 mg/kg cohort, Part A).

^f^ Patients without a postbaseline tumour assessment were considered non-responders.

**Table S4. Summary of profiles and outcomes for patients with NC**

| **Patient** | **Age,  years** | **Sex** | **Translocation** | **Best response** | **Time  on study, days** |
| --- | --- | --- | --- | --- | --- |
| 1 | 54 | M | BRD3-NUT | PR | 115 |
| 2 | 52 | M | NSD3-NUT | PR | 784 |
| 3 | 39 | M | BRD4-NUT | SD | 86 |
| 4 | 33 | M | BRD4-NUT | SD | 64 |
| 5 | 52 | M | BRD4-NUT | SD | 30 |
| 6 | 45 | F | BRD3-NUT | SD | 64 |
| 7 | 47 | M | BRD4-NUT | SD | 309 |
| 8 | 62 | F | ZNF532-NUT | PD | 15 |

Abbreviations: F, female; M, male.


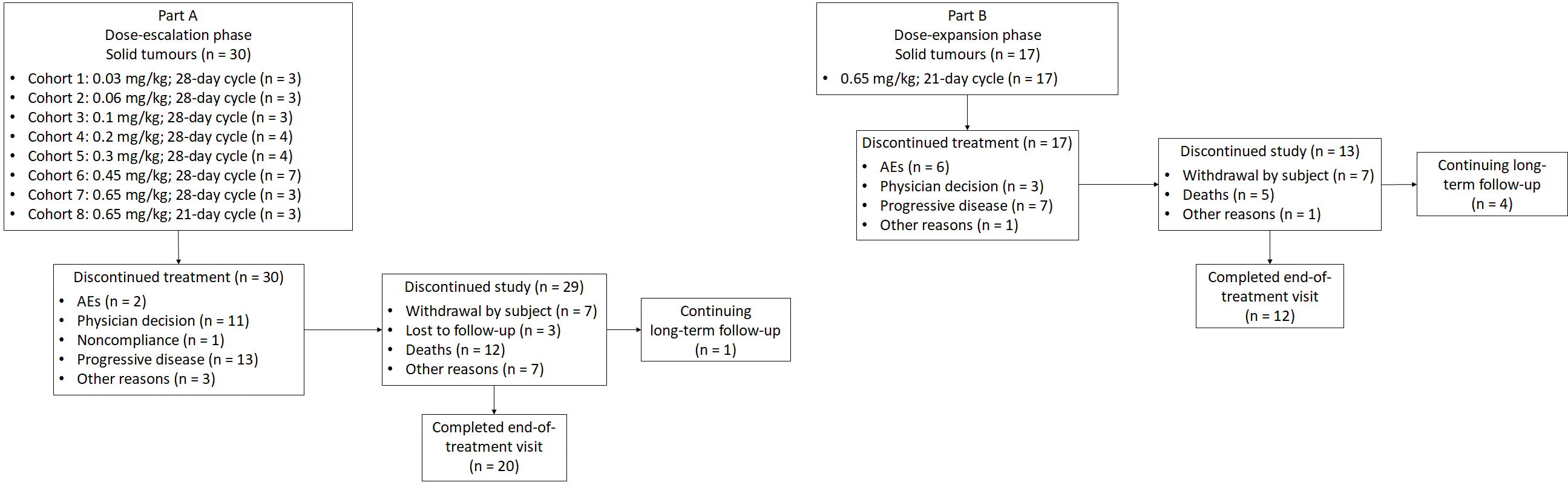


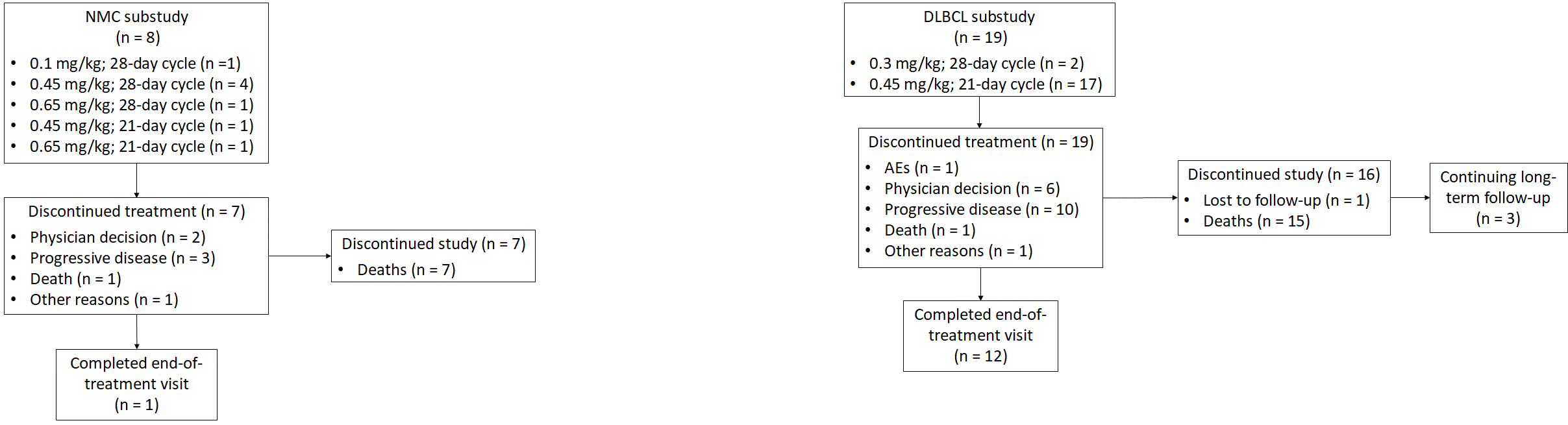


**Figure S1.** Study design and patient disposition.


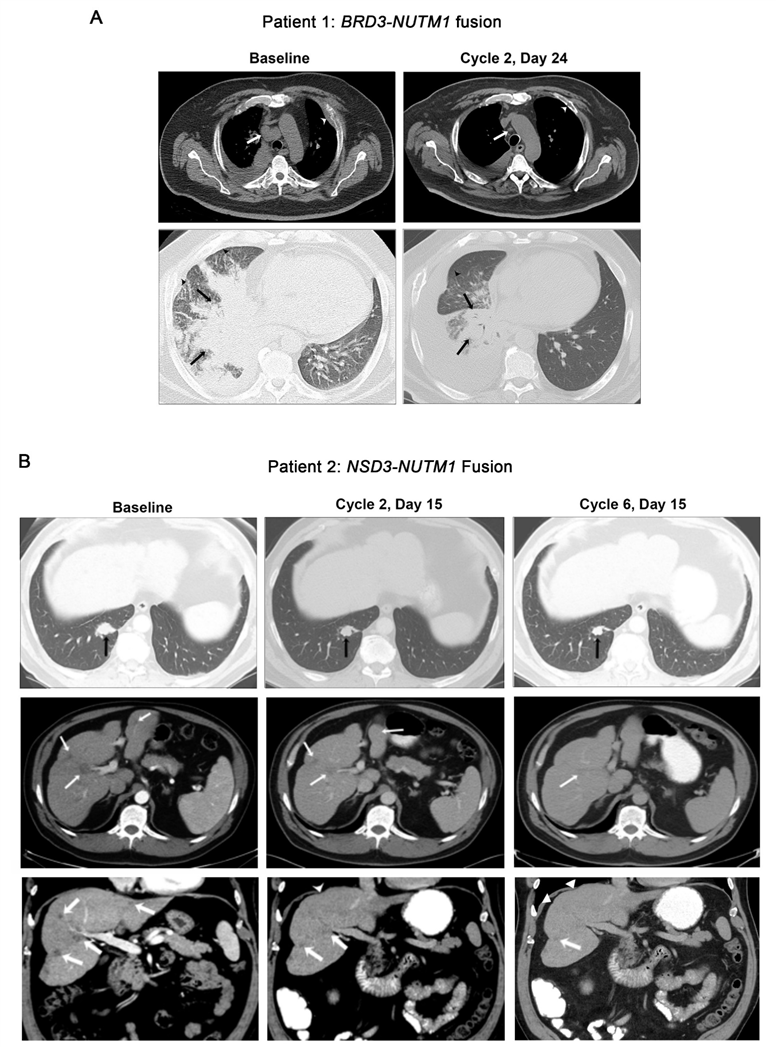


**Figure S2.** Representative RECIST responses in patients with NC. **A,** Computed tomography (CT) images demonstrating a partial response (PR) in a 54-year-old man with tumour harbouring BRD3-NUT rearrangement (Supplementary Table S3, patient 1). Pre-treatment axial CT images in soft tissue and lung windows, respectively, demonstrate precarinal adenopathy (*white arrow*), a left rib lesion with associated soft tissue (*white arrowhead*), a large right lung consolidative opacity (*black arrows*), and interstitial thickening at both lung bases, suggesting lymphangitic spread of disease (*black arrowheads*). Follow-up axial CT images in soft tissue and lung window after 7 weeks of treatment showed interval resolution of mediastinal adenopathy (*white arrow*), the left rib lesion soft tissue (*white arrowhead*), improvement in the right lung mass (*black arrows*), and resolution of lymphangitic carcinomatosis (*black arrowhead*). Per RECIST v1.1, the tumour burden decreased by 49.3%, which was maintained for an additional 8 weeks of RO6870810. **B,** CT images demonstrating a PR in a 52-year old man with tumour harbouring NSD3-NUT fusion (Supplementary Table S3, patient 2). Pre-treatment axial CT images shortly after completion of chemotherapy demonstrated a right lower lobe lung mass (*black arrow*) and several low-density hepatic lesions (*white arrows*). Follow-up CT images toward the end of cycle 2 demonstrated a 22.4% reduction in the size of the lung mass and improvement in the liver lesions. In addition, increased lobular contour of the liver indicates retraction due to fibrosis, also suggesting a response to treatment (*white arrowheads*). A PR was noted after completion of dosing in cycle 4, with a 31.4% reduction in the size of the lung mass and near resolution of liver lesions; this was confirmed after cycle 6, at which time the lung mass was reduced 42.5% from baseline. This response has been maintained through 10 cycles of RO6870810 and is ongoing.

**A**

**
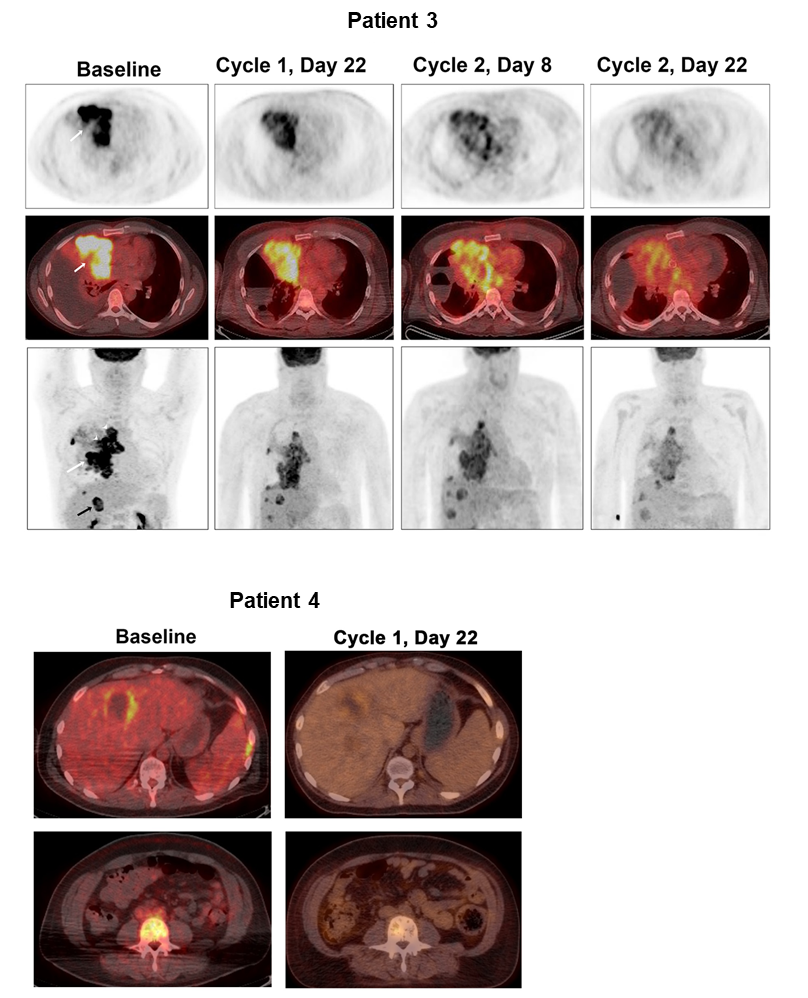
**

**B**

**Figure S3.** Other instances of clinical benefit in the NC population. **A**, ^18^Fluorodeoxyglucose positron emission tomography/CT (^18^F-FDG-PET/CT) findings showing metabolic response in PET images (*upper panels*) and fused axial ^18^F-FDG-PET/CT images and PET images (*middle panels*) at the level of the midchest as well as maximum intensity projection (MIP) PET images (*lower panels*) at baseline prior to RO6870810 and at several time points over the first 2 cycles of study treatment are displayed (Supplementary Table S3, patient 3). Baseline images show intensely FDG-avid, right pleural-based, pleuro-parenchymal disease (*white arrow*), with right pleural lesions mimicking liver metastases (*black arrow*) and lymph nodes at mediastinal and right hilar stations (*white arrowheads*) with a maximal standardised uptake value (SUV_max_) of 12. Follow-up images show a decrease in tracer avidity of the disease, with an SUV_max_ of 6, 5, and 4 at 3, 5, and 7 weeks after initiation of RO6870810, respectively. The patient had a thoracic tumour harbouring BRD4-NUT rearrangement. He had initiated standard chemotherapy with rapid progression prior to RO6870810. He had both clinical and radiographic progression after achieving a metabolic response through the first 7 weeks of study treatment. **B,** Representative baseline and 1-month MIP PET images for another patient who also experienced a transient metabolic response (Supplementary Table S3, patient 4). The patient had a large mediastinal mass with left lung involvement as well as liver and diffuse bone metastases. He was initially treated with palliative radiation followed by carboplatin/etoposide. A few days after initiation of RO6870810, severe chest and bone pain almost completely resolved. After 1 month, disease improvement was observed. His disease progressed after an additional month of treatment.

**
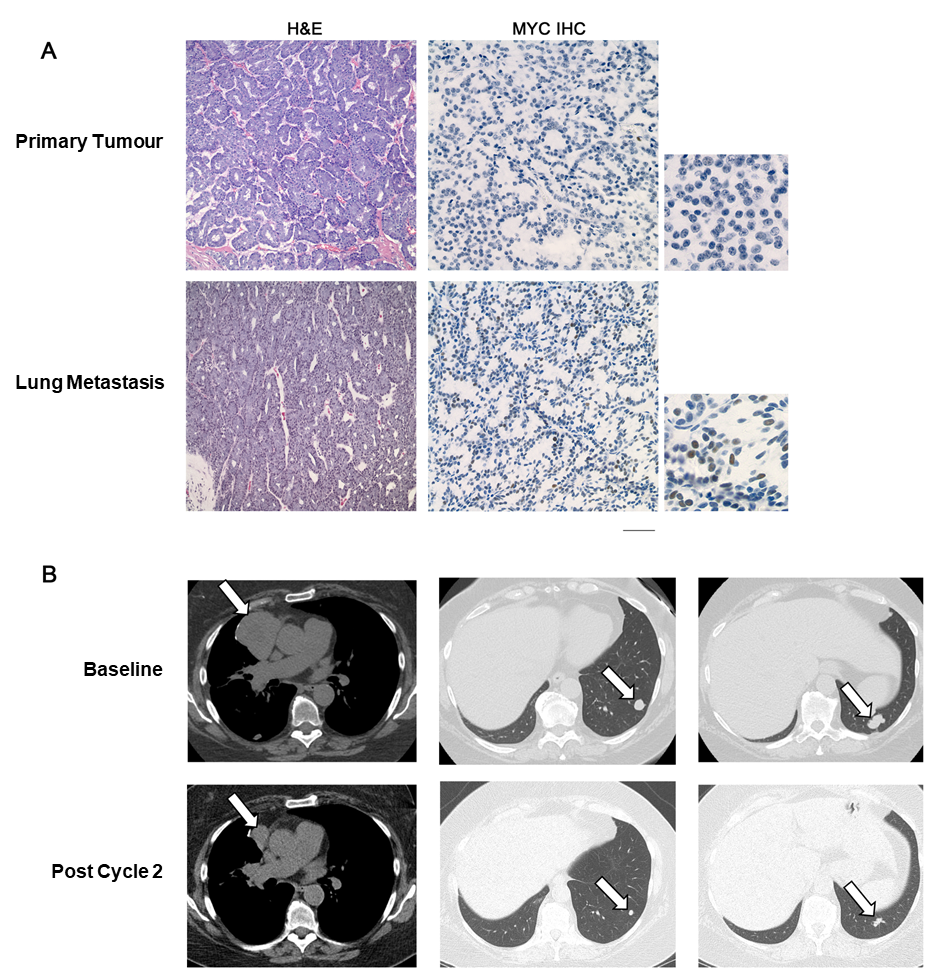
**

**Figure S4.** Representative haematoxylin and eosin (H&E) and c-MYC staining and response in a patient with salivary gland cancer. **A,** Representative sections stained for H&E and c-MYC of the primary tumour and lung metastasis. The primary and metastatic tumours were subjected to targeted NextGen sequencing (409 gene panel) using the ion torrent platform, demonstrating somatic mutations *FBXW7 Q242H*, *FBXW7 S582L*, as well as *KDM6A S763I* and *PIK3C2B Q877_W878K*. These results suggest the possibility of bi-allelic *FBXW7* loss. Although *FBXW7* alterations were present in both the primary and metastatic tumours, only the metastatic tumour expressed Myc, as demonstrated by immunohistochemistry (IHC) performed with a rabbit monoclonal c-Myc antibody (clone Y69). Normal parotid and lung parenchyma were negative for Myc by IHC, as was the primary tumour. The metastasis stained positively in ≈50% of the tumour cells. The expression was restricted to metastatic tumour cells and was nuclear. Bar 70 µM. **B,** Representative lung and mediastinal windows from chest CT images taken prior to and after 2 cycles of RO6870810.

**A**

**
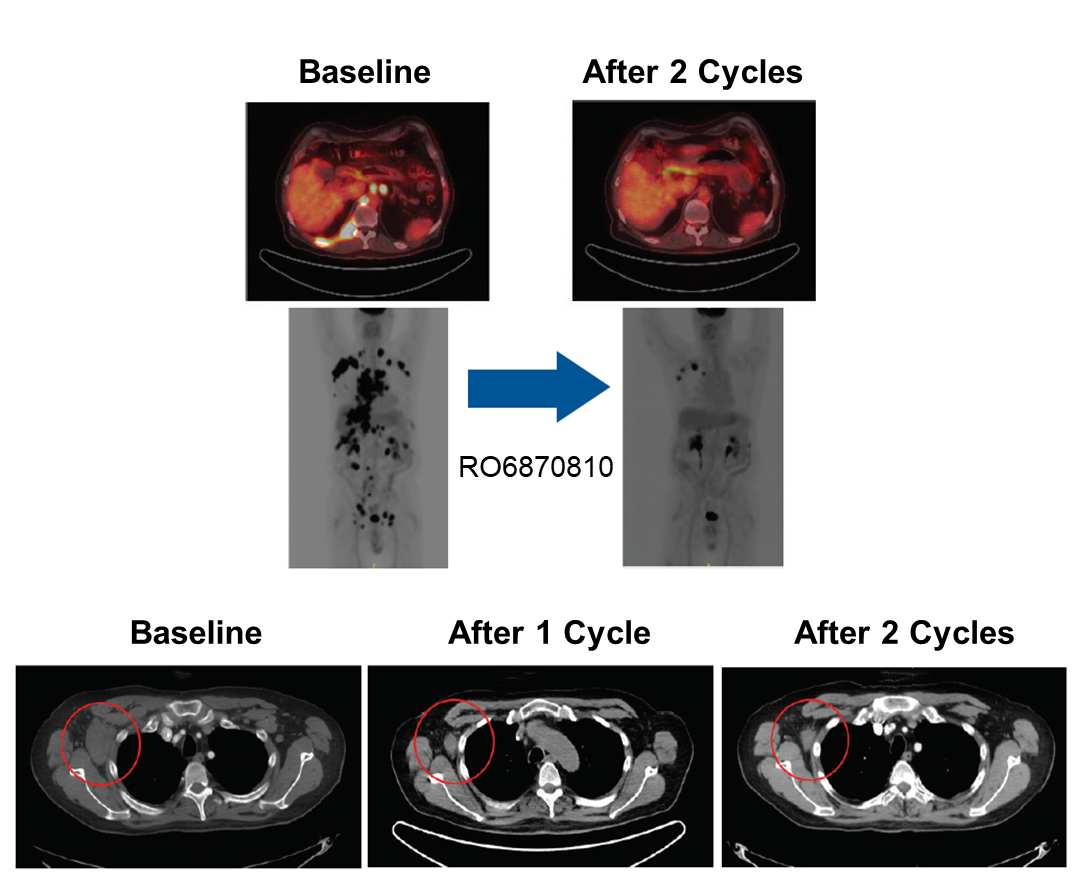
**

**B**

**
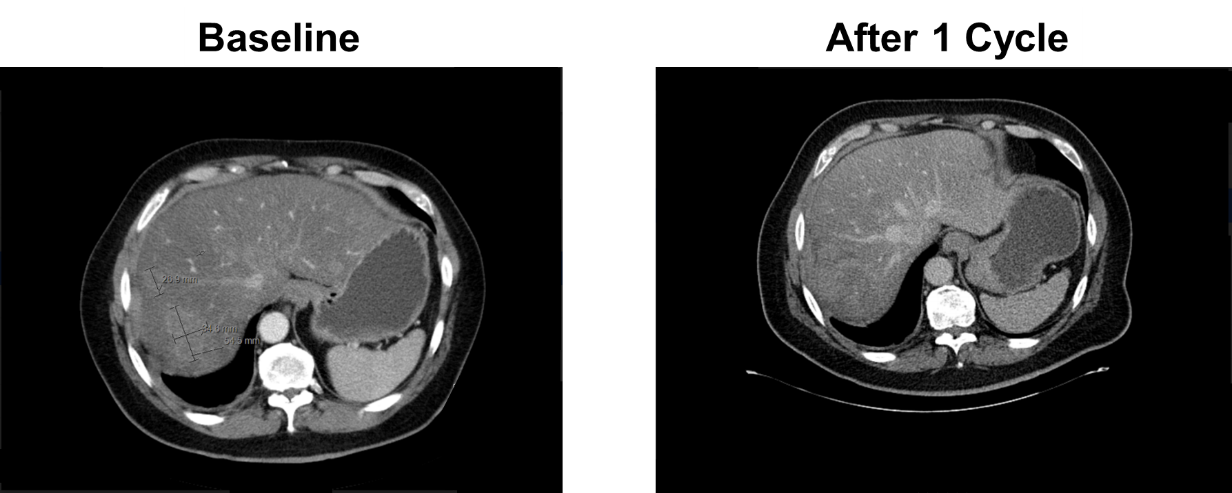
**

**Figure S5.** Representative responses in patients with DLBCL. **A,** ^18^F-FDG-PET, MIP PET, and CT images demonstrating a PR (55% decrease in target tumour volume) after cycle 1 in a 66-year-old man with stage IV disease. **B,** CT images demonstrating a PR after cycle 1 in a 60-year-old man with hepatic involvement.
